# Unveiling the Hidden Dangers of Plasticizers: A Call for Immediate Action

**DOI:** 10.3390/toxics11060527

**Published:** 2023-06-12

**Authors:** Po-Chin Huang, Wei-Chun Chou

**Affiliations:** 1 National Institute of Environmental Health Sciences, National Health Research Institutes, Miaoli 350, Taiwan; 2 Department of Medical Research, China Medical University Hospital, China Medical University, Taichung 404, Taiwan; 3 Research Center for Environmental Medicine, Kaohsiung Medical University, Kaohsiung 701, Taiwan; 4 Department of Environmental and Global Health, College of Public Health and Health Professions (PHHP), University of Florida, Gainesville, FL 326, USA; w.chou@ufl.edu

Over the last several decades, plasticizers have seamlessly integrated themselves into our daily routines, permeating a vast array of commonly encountered products such as food containers, toys, medicines, building materials, electronic devices, cosmetics, perfumes, and personal care items [1,2]. However, recent scientific research has generated growing concerns about the potential health risks associated with these omnipresent substances.

Plasticizers, including phthalates, bisphenols, and their substitutes, have experienced global utilization on an unprecedented scale. Unfortunately, this prevalence has resulted in a worrisome surge in human exposure to these compounds. Studies have unequivocally demonstrated that specific plasticizers possess the capacity to disrupt both animal and human endocrine systems [2,3], leading esteemed scientists and governments in the United States and the European Union to classify them as endocrine-disrupting chemicals (EDCs).

Moreover, emerging evidence derived from mechanistic and epidemiological studies further bolsters the notion that prolonged and low-dose exposure to plasticizers may have deleterious effects on the endocrine, reproductive, and neurological systems, potentially engendering the onset of chronic diseases [4,5,6]. These findings carry profound implications for the health and well-being of diverse populations worldwide.

Furthermore, recent data obtained from human biomonitoring has unveiled elevated levels of plasticizer substitutes within the general population [2]. This alarming revelation necessitates a comprehensive assessment of the potential health impacts posed by these substitutes [7,8]. As we continue to gather more information regarding these emerging compounds, it becomes increasingly imperative to delve into the consequences of their exposure and prioritize public health.

Recognizing the urgency and significance of these findings, this Special Issue endeavors to illuminate the potential health consequences stemming from human exposure to plasticizers and their substitutes. By convening experts from various disciplines, this compilation of research papers seeks to deepen our understanding of the risks associated with these compounds and their potential long-term ramifications. Moreover, it strives to identify strategies for mitigating these risks and promoting the adoption of healthier alternatives.

The scientific community must seize this critical opportunity to explore the implications of plasticizer exposure and raise awareness among policymakers, industry leaders, and the general public. Safeguarding human health necessitates collaborative efforts encompassing interdisciplinary research, regulatory action, and active public engagement.

This Special Issue provides updated knowledge on environmental sources [9,10], mechanistic models [11,12,13], advanced analytical methods [10,14], exposure risk [15], and potential human health impacts [16,17,18] of plasticizers and their substitutes. The inclusion of this valuable and up-to-date information contributes to a deeper understanding of how plasticizers and their substitutes can potentially affect human health.

In conclusion, the pervasive use of plasticizers in our daily lives compels us to conduct a comprehensive examination of their potential health effects. The evidence supporting their classification as endocrine-disrupting chemicals is too substantial to disregard. This Special Issue provides a platform for the scientific community to delve into the risks associated with plasticizers and their substitutes, fostering an informed dialogue, and advocating for measures that protect human health. By addressing this issue proactively, we can pave the way for a safer and healthier future for generations to come.

## Data Availability

Not applicable.
